# Use of TDCS with proprioceptive exercises to improve gait and balance in visually impaired children and preadolescents: a protocol for randomized clinical trial study

**DOI:** 10.3389/fresc.2025.1465846

**Published:** 2025-06-05

**Authors:** Roberta Carneiro de Toledo, Rodolfo Borges Parreira, Deborah Carvalho da Silva Cardoso, Natália de Almeida Carvalho Duarte, Jamile Benite Palma Lopes, Lorraine Barbosa Cordeiro, Daniela Rosana Pedro Fonseca, Iranse Oliveira Silva, Renata Calhes Franco, Karla Cristina Naves de Carvalho, Andrei Machado Viegas da Trindade, Samara Lamounier Santana Parreira, Manuela Galli, Veronica Cimolin, Claudia Santos Oliveira

**Affiliations:** ^1^ Master's and Doctoral Programs in Human Movement and Rehabilitation, Evangelical University of Goiás, Anápolis, Brazil; ^2^ Physical Therapy Department, Evangelical University of Goiás, Anápolis, Brazil; ^3^ Master's and PhD in Health Sciences, Faculty of Medical Sciences, Santa Casa de Misericórdia, São Paulo, Brazil; ^4^ Department of Electronics, Information and Bioengineering, Politecnico di Milano, Milan, Italy

**Keywords:** blindness, transcranial stimulation, gait, child, child development, tDCS

## Abstract

In the absence of information from the visual system, balance is guided by only two of the three afferent systems. If there is no early stimulation of these systems, blind children tend to become passive, which can have a negative impact on muscle tone, coordination and balance. The aim of the present study protocol is to investigate whether transcranial direct current stimulation (tDCS) can enhance the effects of static and dynamic proprioceptive exercises on gait and balance control in children and preadolescents with acquired or congenital visual impairment. This randomized controlled trial will be conducted in three phases, starting with a cross-sectional analysis, followed by a pilot study, and concluding with a full-scale clinical trial. The study will be conducted following approval from the institutional review board of Universidade Evangélica de Anápolis, Anápolis, GO, Brazil (certificate number:4610052.6.0000.5076). The study will be divided into three phases. Phase 1 will be a cross-sectional study to characterize gait, postural control and balance (static and dynamic) in the sample. Phase 2 will be a pilot study that will serve to determine the sample size in Phase 3. Both phases 2 and 3 will employ the same methods and will constitute a randomized, controlled, double- blind, clinical trial. The participants will be randomly divided into four groups: (G1) active tDCS + static proprioceptive exercises; (G2) sham tDCS + static proprioceptive exercises; (G3) active tDCS + dynamic proprioceptive exercises; (G4) sham tDCS + dynamic proprioceptive exercises. The results will be based on evaluations performed on three occasions [preintervention, postintervention (after ten treatment sessions) and 1-month follow-up] and will involve three-dimensional gait analysis as well as assessments of functional mobility functional and balance (static and dynamic). The expected outcomes of this study protocol include determining the postural differences, functional mobility, and static balance between children and pre-adolescents with congenital and acquired visual impairment and enable the establishment of new rehabilitation protocols.

## Introduction

According to the Action Plan of the Vision 2020 Program by the International Agency for the Prevention of Blindness (IAPB), it is estimated that 1.4 million children worldwide have some form of visual impairment (1). The agency reports that the primary causes of childhood blindness include corneal scars, cataracts, glaucoma, retinopathy of prematurity, refractive errors, and low vision, which encompasses untreatable visual impairment and blindness in all regions globally. Spatial orientation and independent mobility without sight present challenges (2, 3). In the absence of visual information, navigation primarily depends on integrating cues related to direction, distance, and speed derived from vestibular and proprioceptive inputs (4, 5). Blind children need to integrate and synthesize information from other senses and must receive consistent and adequate stimulation from an early age to support their typical development without neuropsychomotor deficits (6, 7).

Balance is a result of interactions among the visual, somatosensory, and vestibular systems, which provide feedback to the central nervous system on necessary adjustments. In the absence of visual information, balance is guided by only two of these three afferent systems (7). Thus, visually impaired individuals rely more heavily on vestibular and somatosensory information to maintain balance, whereas sighted individuals primarily rely on visual stimuli (8). To maintain balance and avoid falls, visually impaired individuals exhibit postural deviations from childhood through adulthood and adopt compensatory postures to maintain an upright stance, which influences gait patterns (9, 10). Blind individuals display behavioral strategies in the postural control system that produce increased body sway, enhancing afferent information from remaining senses (11). Interestingly, when blind children are stimulated with proprioceptive exercises, postural control improves, as demonstrated by reductions in sway velocity and displacement of the center of pressure (12).

That low vision in children aged eight to eleven affected postural stability in the standing position as well as the speed of postural adjustments, negatively impacting balance (13). These findings provide valuable insights into the adjustment mechanisms that contribute to maintaining an upright posture in challenging situations. Houwen et al. (14) found that visually impaired individuals exhibited reduced gait speed, shorter stride length, and longer support phase duration during gait compared to sighted individuals. Exercises that stimulate vestibular and proprioceptive pathways are thus crucial for visually impaired individuals. For instance, proprioceptive exercises can improve balance and favor body stability by reducing center of pressure movements or by enabling faster recovery (15).

Proprioception and sensory information from the plantar surface are essential to the maintenance of postural control under typical conditions for blind individuals (16, 17). Blind children who are not stimulated from an early age tend to become passive, with negative impacts on muscle tone, coordination, and balance. Therefore, early intervention is crucial to prevent potential neuromotor delays in these individuals (18).

The combination of motor therapies with methods that stimulate specific areas of the brain, such as the cerebellum, may yield more effective results than motor therapy alone (17, 18). The cerebellum plays a crucial role in motor coordination and balance control, making it a strategic region for interventions aimed at improving posture and gait. According to Zhou et al. (17), transcranial direct current stimulation (tDCS) influences cortical and cerebellar networks during postural control and gait tasks by modulating cortical excitability, which may enhance motor function during cognitive tasks. Recent studies indicate that cerebellar tDCS also promotes neural plasticity and improves motor control in children with neuromotor disorders (19–22). Parreira et al. (20) demonstrated that tDCS can enhance motor evoked potential (MEP) parameters, correlating with improvements in motor control. The MEP reflects corticospinal neuron excitability (23) and is associated with neural plasticity (23). Furthermore, MEP changes extend beyond areas directly stimulated by tDCS (23, 24). However, the effects of cerebellar tDCS on visually impaired children and preadolescents remain poorly understood, underscoring the need to investigate its potential in improving postural control and motor coordination in this population.

This study, therefore, aims to assess whether tDCS can amplify the effects of static and dynamic proprioceptive exercises on gait and balance in children and preadolescents with acquired or congenital visual impairment. The study seeks to correlate differences in outcomes related to gait, static and dynamic balance, and functional mobility between dynamic and static proprioceptive exercises. It also analyzes the specific effects of these exercises on gait, balance, and functional mobility by conducting a comparative analysis of the effects of active vs. sham tDCS, combined with proprioceptive exercises, on the same variables in children and preadolescents with acquired and congenital visual impairment. Our hypothesis is that tDCS combined with static and dynamic proprioceptive exercises may improve gait and balance, as these individuals exhibit deficits in balance, posture, and gait due to a lack of visuomotor coordination. We believe the proposed protocol could enhance gait, balance, and postural control in these individuals by modulating neuroplasticity (23, 24).

## Materials and methods

### Study design

**Figure 1:**
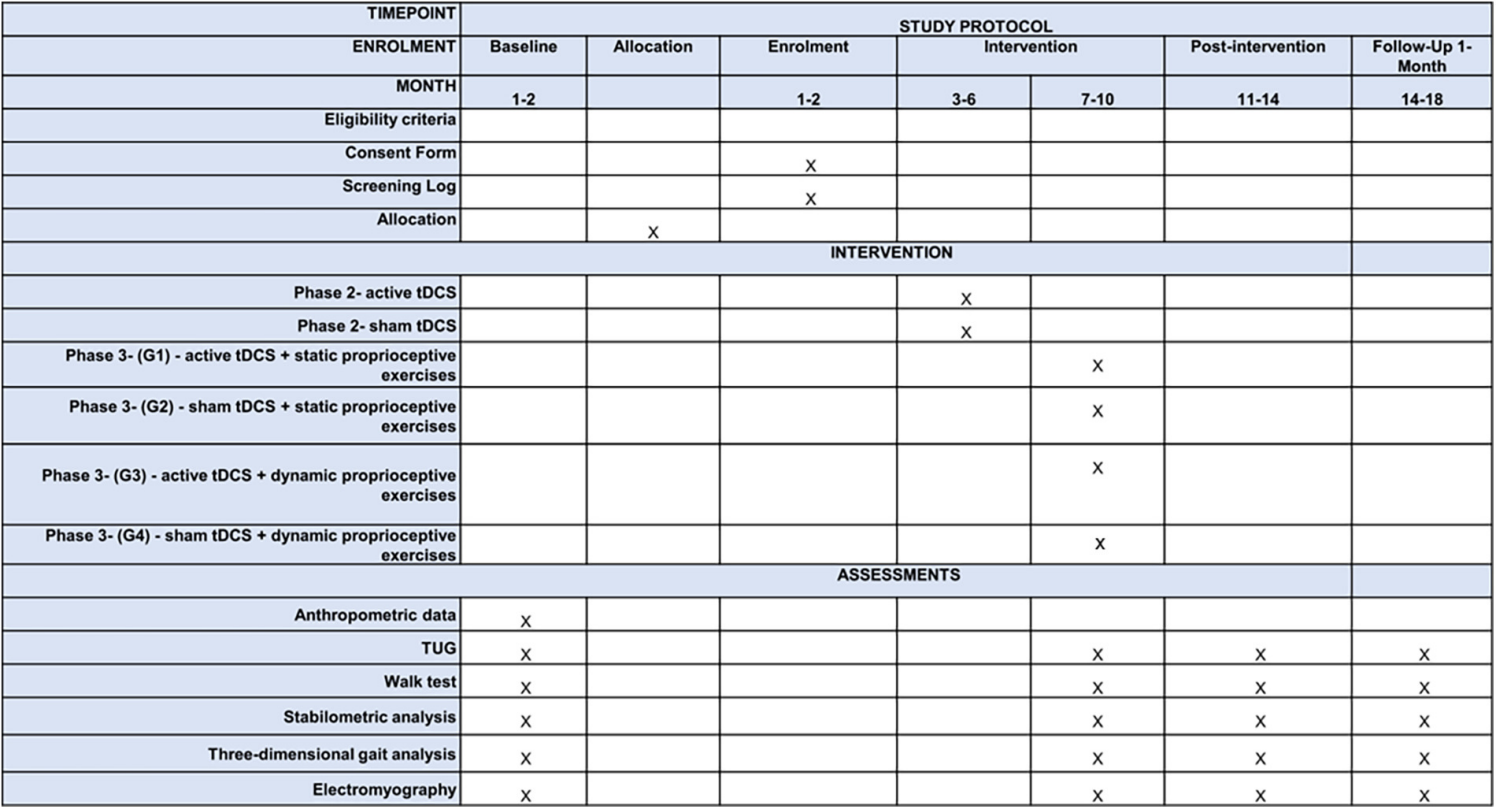
Flowchart of study design based on CONSORT 2010 guidance for protocols of clinical trials. G1 (Group 1), active tDCS combined with static proprioceptive exercises; G2 (Group 2), sham tDCS combined with static proprioceptive exercises; G3 (Group 3), active tDCS combined with dynamic proprioceptive exercises; G4 (Group 4), sham tDCS combined with dynamic proprioceptive exercises; *t*, time in months; *n*, sample number.

This protocol study is a proposal for a clinical trial study that followed the standard protocol items for clinical trials according to SPIRIT 2013 statement (25). The study will be divided into three phases (Table 1). Phase 1: will comprise a cross-sectional study to characterize the sample's postural control, static and dynamic balance, and gait. Phase 2: will comprise a pilot study with a convenience sample of 6-to-12-year-old children and preadolescents with either acquired or congenital visual impairment and children and preadolescents with normal vision. The objective of the pilot study will be to observe the effects of tDCS on postural control and gait during a proprioceptive exercise protocol. The results of this phase will be used to estimate the sample size of Phase 3, described in Figure 1. Phase 3: will be a randomized, controlled, double-blind, and clinical trial in children and preadolescents with either acquired or congenital visual impairment where we will seek if there will be differences among pre-intervention, post-intervention, and after 1 month of treatment throughout the tridimensional gait analysis, electromyography, functional mobility, and static and dynamic gait assessment.

**Table 1:** Phases, outcome measures, and evaluation of the protocol study.

Phase	Objective	Participants	Primary outcome measures	Secondary outcome measures	Evaluation times
Phase 1: cross- sectional characterization	To characterize gait, balance, and postural control before the intervention.	Children and preadolescents with visual impairment (acquired or congenital blindness) and sighted children (control group).	- Three-dimensional gait analysis (gait speed, stride length, support phase, COP displacement) - Static and dynamic balance assessment - Timed Up and Go (TUG) test - Pediatric Balance Scale (PBS)	- Pediatric Evaluation of Disability Inventory (PEDI)	Baseline assessment (pre- intervention)
Phase 2: pilot study	To assess the feasibility of the protocol and determine the sample size for Phase 3.	Convenience sample of children with acquired or congenital visual impairment.	- Three-dimensional gait analysis - Static and dynamic balance assessment - Timed Up and Go (TUG) test - Pediatric Balance Scale (PBS)	- Electromyography (EMG) - PEDI - G-sensor® analysis for spatiotemporal gait parameters	- Pre-intervention - Post-intervention (after 10 sessions) - 1-month follow-up
Phase 3: randomized controlled trial (RCT)	To test the effectiveness of tDCS combined with static and dynamic proprioceptive exercises on gait, balance, and functional mobility.	Children and pre-adolescents with visual impairment, randomized into 4 groups: G1: Active tDCS + static exercises G2: Active tDCS + dynamic exercises G3: Sham tDCS + static exercises G4: Sham tDCS + dynamic exercises	- Three-dimensional gait analysis - Static and dynamic balance assessment - Timed Up and Go (TUG) test - Pediatric Balance Scale (PBS)	- Electromyography (EMG) - PEDI - G-sensor® analysis for spatiotemporal gait parameters	- Pre-intervention - Post-intervention (after 10 sessions) - 1-month follow-up

Timeframe of development of study. G1 (Group 1), active tDCS + static proprioceptive exercises; G2 (Group 2), sham tDCS + static proprioceptive exercises; G3 (Group 3), active tDCS + dynamic proprioceptive exercises; G4 (Group 4), sham tDCS + dynamic proprioceptive exercises; *t*, time in months.

### Ethical approval

The proposed study will be conducted following the guidelines and regulatory norms stipulated by the National Board of Health in October 1996 and updated in Resolution 466 of 2012 governing research involving human subjects in Brazil. The study will be conducted following approval from the institutional review board of *Universidade Evangélica de Anápolis*, Anápolis, GO, Brazil (certificate number: 4610052.6.0000.5076). The study protocol was registered in the *Registro Brasileiro de Ensaios Clínicos* (ReBEC) (number RBR-3chg6v5) available in https://ensaiosclinicos.gov.br/.

### Sample size and recruitment

The sample size for Phase 3 of the study will be estimated based on the minimal difference between the mean of the analysis of variance results obtained from both gait speed and the displacement of the center of pressure (COP) as the primary outcome obtained in the groups of the pilot study (Phase 2) considering the primary outcome. Thus the sample size will be estimated with a unidirectional alpha of 0.05 and an 85% statistical power. The sample determined by the calculation will be increased by 20% to compensate for possible dropouts. Since this is a proposal study for a clinical trial, for the part of the study, the main objective of the study will be to establish an effect size for tDCS treatment in association with proprioceptive exercises. This is independent of the number of volunteers but allows us to estimate the number that would be required to achieve statistical significance depending on the effect size we observe.

Children and preadolescents with acquired or congenital visual impairment will be sent by healthcare providers of the *Centro Municipal de Atendimento à Diversidade* [CEMAD (Municipal Diversity Care Center)] in the city of Anápolis, Brazil. Preselected individuals will then be screened, with the collection of personal information and anthropometric measures. Congenital and acquired blindness will be characterized based on the classification of the degree of visual impairment proposed by the World Health Organization, the International Statistical Classification of Disease, and the 10th Edition of the International Classification of Disease, in which visual acuity <20/400 or <20/200 in the better eye is classified as visual impairment (26, 27).

The study will be divided into three phases. The results of Phase 2 will serve as the basis for Phase 3. Phase 3 was designed according to updated guidelines for reporting parallel group randomized trials CONSORT, 2010 (28). A convenience sample will be used in the first phase, composed of 10 male and female children and preadolescents with acquired or congenital visual impairment between 6 and 12 years of age, who will be recruited from CEMAD in the city of Anápolis, and a control group of 10 sighted male and female children and preadolescents in the same age range, who will be recruited through informal invitations. The visually impaired patients will be referred with a medical diagnosis performed by an ophthalmologist. These 20 individuals will be used to characterize the gait pattern, balance, and functional mobility. The second phase will be a pilot study conducted with the same methods as the main study and will provide data for the calculation of the sample size (Figure 1). To determine differences in gait pattern, balance, and functional mobility subjects with blindness will perform the assessment and tests with/without a guide stick and when wearing shoes and while barefoot.

As this research involves children and pre-adolescents, parents and/or guardians will be consulted in advance to clarify the research objectives, as well as risks and benefits, and those who accept to participate in the research will sign a consent form through the Adult Consent Form subscription. Children and pre-adolescents will also be informed about the objectives and purposes of the research, which, if they also agree, must sign the Child Assent Form developed in a specific language for the age group. At the end of the study, subjects from the sham group will receive active tDCS as a form of treatment and adherence to the study. Finally, if a patient decides to withdraw from the follow-up, the reasons for the withdrawal will be recorded for the subsequent analysis in the interpretation of the results.

### Eligibility criteria

To be included in the study, children and preadolescents must have any abnormalities of the visual system that let them to a total blindness such as: retina disorders, glaucoma, macular degeneration, retinitis pigmentosa, toxoplasmosis, cataracts, detached retina, abnormalities of the optic nerve, Leber's amaurosis, and astrocytoma. For exclusion criteria we will set for: use of medication affecting the central nervous system, balance or coordination, symptoms of vertigo or dizziness, clinical condition affecting balance and gait, surgery of the lower limb, vascular and sensory disease. Additional exclusion criteria that will be considered for tDCS are: frequent migraine/headache, metallic implant in the head or neck, scalp or skin condition and seizures.

### Randomization and allocation to groups

Randomization will take place in blocks. Participants and investigators will be blind to the tDCS condition allocation. Individuals who meet the eligibility criteria will be randomly allocated to one of the four study groups using a digital randomization platform (http://www.randomization.com). An opaque and sealed envelopes will be used to conceal group information. After signing the informed consent, individuals will choose an envelope with the name of the group to which they will be assigned in phases 2 and 3. This step will be managed by a third person who is not part of the study. The groups will be divided according to the type of therapy: Group 1 (G1)—active tDCS combined with static proprioceptive exercises; Group 2 (G2)—sham tDCS combined with static proprioceptive exercises; Group 3 (G3)—active tDCS combined with dynamic proprioceptive exercises; Group 4 (G4)—sham tDCS combined with dynamic proprioceptive exercises.

#### Masking

Participants, therapists, and assessors involved in the study will be blinded to the treatment allocation. During the intervention, both active and sham tDCS conditions will have the electrodes applied in the same manner. However, in the sham condition, the stimulation will only be applied for the first 30 s to mimic the sensation of tDCS, after which no current will be delivered for the remaining session time.

The therapists administering the intervention will not have access to the allocation sequence and will perform the same proprioceptive exercises for all participants, regardless of the group. To prevent any bias, the individuals responsible for collecting and analyzing the data will also be blinded to group allocation.

#### Measures

##### Timed up and go test

The Timed Up and Go (TUG) test is widely used to assess functional mobility and consists of the time in seconds required to stand up from a standard chair, walk along a straight line for three meters, turn around, walk back to the chair, and sit down again. A longer time required to complete the task denotes poorer functional mobility. At the beginning of the test, the participant will be seated with the back against the backrest of the chair and must return to this same position at the end of the test. The test will begin with the command “Go”. The TUG test will be performed four times under the following conditions: in shoes with a cane; in shoes without a cane; barefoot with a cane; and barefoot without a cane. The participants will first be given a practice run to become familiar with the test. During the test, the G-sensor (BTS Bioengineering) will be used for the precise quantification of the time required to perform the task (30). We will analyze functional mobility throughout the variables such as: phase duration (s), acceleration, and velocity of the TUG, sit-to-stand, stand-to-sit, mid-turn, and end-turn.

##### Walk test

For the walk test, the participant will be instructed to walk along a straight line for seven meters. The test will be performed with an inertial sensor (G-Sensor®), which will collect spatiotemporal variables, general kinematic variables, symmetry index, propulsion index, and pelvic kinematics (31). Participants will perform the test three times and the mean of the trials will be used for statistical analysis.

##### G-Sensor

The G-Sensor® (BTS Bioengineering S.p.A. Italy) (32, 33) is a portable, wireless system of inertial sensors for human movement analysis. The device will be held in place by a specific strap, which will enable the participant complete freedom for walking, running, and jumping. The sensor sends all data to a computer via Bluetooth. At the end of each analysis, a detailed report is furnished on all variables recorded during the test. The sensors are controlled by a data recording unit (up to 16 elements) through ZigBee radio communication. Each sensor measures 62 × 36 × 16 mm, weighs 60 g, and is composed of a three-axis accelerometer (maximum scale: ±6 g), three-axis gyroscope (complete scale: ±300°/s) and three-axis magnetometer (complete scale: ±6 Gauss). The device is calibrated with the acceleration of gravity immediately after its fabrication. In the present study, the device will be used during the execution of the TUG test and Walk Test to obtain precise data on spatiotemporal variables and functional mobility. The data from the inertial sensor will be transmitted via Bluetooth to a computer and processed using the appropriate software (BTS G-STUDIO, version 2.6.12.0), which automatically furnishes the variables (32, 33).

The following information will be collected: stride length (m); gait speed: mean instantaneous velocity within the gait cycle (m s^−1^); cadence (number of steps per minute [steps min^−1^]; position and duration of the swing phase [expressed as the percentage of the gait cycle—the proportion of a gait cycle that involves the stance and swing phases (from toe off to heel contact of the same foot)]; duration of double support (duration of stance phase with both feet, expressed a percentage of the gait cycle); pelvic angulation (tile, obliquity and rotation) of stride (distance between two consecutive heel contacts).

##### Pediatric evaluation of disability inventory (PEDI)

The functional performance will be evaluated quantitatively using the Pediatric Evaluation of Disability Inventory (PEDI), which is a questionnaire administered in interview form to a caregiver with information on the child's performance regarding routine activities and tasks. The test is composed of three parts: the first part addresses skills in the child's repertoire grouped into three functional domains: self-care (73 items), mobility (59 items), and social function (65 items). Each item is scored either 0 (the child is unable to perform the activity) or 1 (the activity is part of the child's repertoire of skills). The scores are totaled per domain (34).

##### Pediatric balance scale (PBS)

The Pediatric Balance Scale (PBS) is a modified version of the Berg Balance Scale, specifically designed to assess balance in school-aged children with mild to moderate motor impairments (35). In this protocol study, we employed the Brazilian version of the PBS, which has been translated and culturally adapted for Brazilian populations (35). The PBS consists of 14 tasks that simulate daily living activities, each scored on a 5-point ordinal scale from 0 to 4, where: 0 = unable to perform the task independently; 1 = requires maximal assistance; 2 = requires moderate assistance or close supervision; 3 = minimal assistance or supervision; 4 = able to complete the task independently. The total score ranges up to 56 points, with higher scores reflecting better balance performance.

Below is a detailed description of the 14 tasks: Sitting to Standing; Standing to Sitting; Transfers (from sitting to lying); Standing Unsupported (up to 2 min); Standing with Eyes Closed (up to 10 s); Standing with Feet Together (up to 10 s); Standing with One Foot in Front (Tandem Stance); Standing on One Leg (up to 10 s); Turning 360 Degrees; Turning to Look Behind; Picking Up Object from Floor; Placing Alternate Foot on Step (Step-ups); Reaching Forward with Outstretched Arm While Standing; and Standing Unsupported, One Foot in Front on a Line (heel-to-toe stance) (36, 37). For this measure, all subjects will perform the PBS in barefoot conditions.

##### Assessment of balance with proprioceptive disturbance

The assessment of static balance with proprioceptive disturbance will be performed using the SMART-D 140® system (BTS Engineering) with two Kistler force plates (model: 9286BA). The acquisition frequency will be 100 Hz and the force will be captured by four piezoelectric sensors measuring 400/600 mm positioned at the extremities of the force plate. The data will be recorded and interpreted by a software program (SWAY; BTS 161 Engineering) integrated and synchronized with the SMART-D 140® system. The participants will be instructed to remain in a static standing position with arms alongside the body and head in the vertical position. Three measurements (45 s each) will be performed to collect postural balance of COP (95% confidence ellipse area; velocity, and RMS in anteroposterior and mediolateral directions) under four different conditions: static balance with proprioceptive disturbance (soft surface) and eyes open; static balance with proprioceptive disturbance and eyes closed; static balance on a firm surface with eyes open; and static balance on a firm surface with eyes closed.

##### Three-dimensional gait analysis and electromyography

Gait will be analyzed along a track measuring 90 centimeters in width by five meters in length. Gait analysis will be performed with the aid of the SMART-D 140® system (BTS Engineering), consisting of eight cameras sensitive to the infrared spectrum synchronized with a video system and computer (SMART-D INTEGRATED WORKSTATION® with 32 analog channels). Two force plates (Kistler, model 9286BA) will be used for the collection of the kinematic gait data, recording displacements of the center of pressure and contact time between the foot and surface of the force plate (38, 39). Therefore, a protocol was implemented whereby bone landmarks were located by manual palpitation by the principal investigator. Reflective markers were firmly attached to the skin with double-sided tape. During the analysis, the participants will be wearing bathing suits. Markers will be positioned according to the protocol described by Davis (39). A total of 22 spherical markers will be placed—three on the trunk, three on each thigh, three on each shin, and two on each foot.

Information will be collected on body mass, height, distance between anterior iliac crests posteriorly, leg length, knee diameter, and ankle diameter. After placing the markers, participants will be instructed to walk along the track with the two force plates positioned in the center. Upon stepping on the force plates, kinematic gait data will be collected and calculated by the video system (BTS, Milan, Italy) synchronized to the data collection system. The following indices will be analyzed based on the spatiotemporal variables (velocity, cadence, step length, stride length, stance phase, and swing phase) and joint angles at specific moments of gait (pelvis inclination, hip flexion-extension, knee flexion-extension, and ankle dorsiflexion-plantar flexion), as well as foot progression.

The electrical activity of the muscles will be collected simultaneously with the three-dimensional analysis of the gait by the electromyograph FREEEMG® (BTS Engineering) composed of eight amplifiers of bioelectric signals, bipolar electrodes with a total gain of 2,000x at a frequency of 20–450 Hz and data transmission wireless. The impedance and common mode rejection ratio of the equipment is >1,015 Ω//0.2 pF and 60/10 Hz 92 dB, respectively. The electrodes will be placed on the motor point of the muscles after cleaning the skin with 70% alcohol to reduce bioimpedance, following the guidelines of the Surface Electromyography for the Non-Invasive Assessment of Muscles (SENIAM) (40). All electromyographic data will be collected and digitized at 1,000 frames/second using the BTS MYOLAB® software program. The electromyographic data will be collected simultaneously with the kinematic readings and both sets of data will be managed by the BTS® system and Smart Capture® software. The electrodes will be attached to the rectus femoris, tibialis anterior, biceps femoris, and soleus muscles bilaterally. In total, a minimum of five walking attempts will be recorded. Of these five attempts, three readings will be considered three times so that the participant becomes familiar with the protocol. To ensure patient safety during the walk, a researcher will be responsible for directing patients through verbal stimuli.

To minimize potential bias, all standardized assessment protocols are designed to ensure accurate and reliable collection. In addition, several training sessions will be offered to assessors and therapists to ensure standardized treatment, assessment, and data analysis. The amount of training will depend on the raters' and therapists' familiarity with clinical scales and treatment techniques. Standard procedures should be followed during evaluation and treatment. There will be several meetings where the Principal Investigator will be briefed on current events and available for consultation with any questions or concerns. Although we attempted to blind patients, therapists, and raters, it is unlikely that patients and therapists would remain blinded during the course of this study due to the nature of the treatment applied. However, to ensure that the risk of bias remains low, patients will be registered in the database via a patient identification code so that raters are blinded during analysis. Only the principal investigator will be aware of the allocation.

#### Outcome measures

##### Primary outcome measures

The primary outcomes focus on quantifying improvements in gait, balance, and functional mobility:

Instrumental Assessments:

1. Three-dimensional Gait Analysis:
  ○ Gait speed (m/s)
  ○ Stride length (m)
  ○ Duration of the support phase (percentage of gait cycle)
  ○ Ground reaction force
2. Balance (force platform):
  ○ COP displacement along the *X* (anteroposterior) and *Y* (mediolateral) axes: Analyzed during static balance tasks performed with proprioceptive disturbance (soft and firm surfaces).

Clinical Assessments:

1. Timed Up and Go (TUG) Test:
  ○ Time (seconds) to complete the TUG test under various conditions (with and without a guide stick, with and without shoes) to assess functional mobility.
2. Electromyography (EMG):
  ○ Muscle activity (µv)
3. Pediatric Balance Scale (PBS):
  ○ Assesses postural control with 14 tasks scored from 0 (unable to perform) to (independently performs). Maximum score: 56 points.

##### Secondary outcome measures

The secondary outcomes aim to provide additional insights into the overall impact of the intervention on daily function and postural control.

Instrumental Assessments:

1. G-sensor® Analysis:
  ○ Assesses spatiotemporal gait parameters (e.g., cadence, step length, propulsion index) using a portable wireless G-sensor® device during walking tasks.

Clinical Assessments:

1. Pediatric Evaluation of Disability Inventory (PEDI):
  ○ Evaluates functional capabilities in three domains: self-care, mobility, and social function. Each item is scored from 0 (unable) to 1 (able).

#### Evaluation protocol

Evaluations will be performed in all phases of the study. Evaluations will be conducted before the intervention, immediately after the intervention, and 1 month after the end of the intervention.

#### Intervention protocol

The therapeutic intervention will consist of ten sessions of transcranial direct current stimulation, which will be applied using the Transcranial Stimulation device (Transcranial Technologies, USA) with two sponge (non-metallic) electrodes measuring 5 × 7 cm moistened with saline solution (41). Stimulation will be administered simultaneously during the protocols.

Each session will have a duration of 30 min, with the same session length for both active and sham stimulation protocols, ensuring a valid comparison. During the ten training sessions, tDCS will be administered with the anode positioned centrally over the cerebellum and the cathode positioned in the central supraorbital region. For sham stimulation, the electrodes will be positioned in the same way and the stimulator will be switched on for the first 30 s, giving the participant the initial sensation of stimulation, but no electrical current will be delivered for the remainder of the session. This is a valid control procedure for studies involving tDCS. In the active groups, a current of 1.5 mA will be administered for the stimulation of the cortices during the 20 intermediate minutes of each session. The stimulation device will automatically and gradually increase to 1.5 mA at the onset of treatment and gradually diminish in the final 10 s.

### Static proprioceptive exercises

The static proprioceptive exercises will be performed on an unstable surface using a balance board (diameter of 40 cm and a height of 10 cm), allowing for movement in both the anteroposterior and mediolateral directions. The static exercises will also include standing on toes with feet apart, standing on toes with feet together, standing on the right foot alone without support, standing on the left foot alone without support, and performing a tandem stance (standing with one foot in front of the other in a straight line). Each exercise will be performed for 30 s, repeated in six sets, with one-minute rest intervals between sets. These exercises will be conducted barefoot to optimize proprioceptive feedback.

### Dynamic proprioceptive exercises

The dynamic proprioceptive exercises involve a series of functional tasks designed to challenge balance and motor coordination. These exercises include (1) walking slowly and then quickly on a mini-trampoline (diameter of 100 cm and a height of 20 cm, providing an unstable surface that engages the vestibular and proprioceptive systems); (2) Walking forward and backward placing one foot immediately behind the other (balance beam measures: 3 meters in length, 10 cm in width, and 15 cm in height from the floor); (3) Stair-climbing exercises, using a staircase with five steps, where each step measures 20 cm in height and 30 cm in depth; (4) Participants will perform exercises while seated on a 65-cm exercise ball, such as anteroposterior and laterolateral movements, circumduction, and bouncing. Exercises involving the mini-trampoline, beam, and stairs will be conducted in three sets of 1 min each, with 30-second rest intervals. The ball exercises will be performed in sets of 30 s, with one-minute rest intervals. Both protocols will be performed in a specially prepared room suitable for individuals with visual impairment in a space measuring approximately 8 × 5 m free of furniture with the ideal temperature, light, and sound. All participants will perform the exercises barefoot wearing clothing suitable for the practice of physical activity. Throughout the intervention, the participant will be accompanied by two physiotherapists to avoid imbalances and falls.

#### Adverse effects

Given that both tDCS and proprioceptive exercises are non-invasive, the likelihood of serious adverse events is low. However, we will constantly monitor for any potential side effects throughout each session. tDCS is generally well tolerated, but participants may experience mild side effects such as tingling or itching sensations at the electrode sites, headaches during or after stimulation, fatigue or drowsiness, and minor skin irritation or redness at the electrode placement areas. To manage these potential issues, participants will be instructed to report any discomfort immediately. If necessary, the session can be paused, and adjustments will be made. For skin irritation, the placement of electrodes can be adjusted, or the sponges can be moistened further. Headaches or fatigue can typically be managed with rest, and participants will be advised to drink water and rest if symptoms persist. The proprioceptive exercises, particularly the dynamic ones, carry a small risk of physical discomfort or injury, such as muscle soreness or temporary imbalance, especially during more challenging tasks like tandem stance, walking on the balance beam, or climbing stairs. To prevent falls or injuries, two physical therapists will be present to supervise each session and provide support. If a participant experiences dizziness or loss of balance, they will be allowed to rest, and the intensity of the exercises can be adjusted according to their ability. If participants report any discomfort during the evaluations, adjustments will be made to the protocol or equipment as necessary, and the session can be paused for the participant's comfort.

#### Status and timeframe of the study

The timeframe of the study is given in Table 1.

#### Statistical analysis

To test the normality of the data we will use the Shapiro–Wilk test and Levene's test for the homogeneity of variances. When data is normally distributed and the assumption of homogeneity will not be violated, parametric analyses will be conducted. When one assumption will not be met, non-parametric tests will be employed or a log transformation of the distribution will be applied. Effect size (Cohen *d* estimate) 23 on PLP changes for within-group and between groups (tDCS vs. MT group) comparisons were calculated. The effect size (Cohen *d* estimate) (41) will be calculated based on the difference between the means of the pre-intervention and post-intervention assessments and presented with their respective 95% confidence intervals based on mean and standard deviation.

In order to find differences between groups (blind and sighted subjects) in Phase 1, a independent *t*-test or a Mann-Whitney test for non-parameteric distribution will be employed to investigate the variable of 3D gait analysis, balance (force platform), TUG test, and PBS as primary outcome and EMG, and PEDI as secondary outcome. For phases 2 and 3, to analyze the primary outcomes a mixed model ANOVA (Analysis of Variance) with repeated measures will be employed, where the fixed factors will be groups (with four levels) and time (pre-intervention, post-intervention, and 1-month follow-up). Partial eta-squared (*η*²*p*) will be used to calculate effect sizes and determine the magnitude of the observed differences. Non-parametric tests will be considered if necessary.

We will implement an interim analysis plan in this study to assess the accumulated data at specified time points before the completion of the study (phase 2). The interim analysis aimed to evaluate the study's primary outcome measures and to make informed decisions regarding the continuation, modification, or termination of the trial. The interim analysis was pre-planned and conducted according to a predefined statistical analysis plan. This approach allowed for an ongoing assessment of the study's effectiveness and safety while ensuring the integrity and validity of the final results. In case of missing data, participants will be simply excluded from the analysis to avoid bias. All tests will use the *p*-value < 0.05. The data will be organized and tabulated using Statistical Product and Service Solutions (SPSS, v.19.0).

## Discussion

The proposed protocol aims to investigate the combined effects of transcranial direct current stimulation (tDCS) and proprioceptive exercises on gait, balance, and functional mobility in children and preadolescents with acquired or congenital visual impairment. Although previous research suggests that tDCS can modulate neuroplasticity and enhance motor control, and that proprioceptive exercises are effective in improving balance and postural stability, the combination of these two interventions has not been extensively studied in the pediatric population, particularly in those with visual impairments. What is known is that interference in the visual system generates a direct impact on the maintenance of balance and gait (40, 41). Low vision can exert negative impacts on sensory-motor, cognitive, and language development in children. These children use their residual vision to explore the environment and need to use their vision in the best way possible to compensate for the impairment (42, 43). Schmid et al. (44) found that the stimulation of different sensory systems through cerebral plasticity early in life was unable to replace normal vision.

Moreover, blind individuals exhibit greater body sway, likely as a way to increase afferent information from the remaining senses. While the effects of proprioceptive exercises on balance are well documented, the proposed study seeks to establish whether the neuromodulatory effects of tDCS can enhance the efficacy of these exercises. We expect that proprioceptive exercises, when combined with active tDCS, will show greater improvements than with sham stimulation. These improvements might be reflected in the fine-tuning of postural control mechanisms and greater activation of motor pathways, which could contribute to more pronounced and lasting effects on balance and gait performance.

According to Bennett et al. (45), blindness exerts an impact on gait, leading to a slower walking speed, shorter stride length, and restricted plantar flexion. An altered gait pattern in the absence of sight has been interpreted as a more cautious walking strategy to avoid falls and accidents. Other studies found that blind individuals with better gait performance were those who reported greater levels of physical activity (14, 46). Moreover, a better performance regarding locomotion may be related to better localization and orientation skills. The systems that participate in the maintenance of balance are vision, touch, proprioception, and the vestibular system (45).

Therapies that provide proprioceptive and vestibular stimulation are of fundamental importance for blind individuals (47). In recent decades, a growing number of studies have evaluated the short-term and long-term effectiveness of noninvasive brain stimulation techniques for various health conditions, with increasing interest in the use of tDCS as a facilitator of neuroplasticity (21, 22, 47, 48). Transcranial direct current stimulation is simple, relatively inexpensive, and can be administered in combination with other cognitive and motor training protocols (49). Studies have demonstrated the effectiveness of tDCS for different neuropsychiatric disorders in adults (48). However, few studies have been conducted with children and adolescents (50). If the expected outcomes are confirmed, the results of this protocol study could have significant implications for the field of pediatric neurorehabilitation, particularly for children with visual impairments. Establishing the efficacy of this combined intervention could lead to the development of more effective, low-cost, and easily implemented rehabilitation protocols. From a broader perspective, the findings of this study could encourage future research on the long-term benefits of combining neuromodulation techniques with traditional rehabilitation exercises. This could include exploring the most effective exercise regimens, and the extent to which these interventions can prevent declines in motor function or mitigate the challenges associated with visual impairment.

### Limitations of study

It is important to recognize that, as this is a proposed protocol, all hypotheses remain speculative until the study is completed. The outcomes, though promising in theory, may be influenced by various factors, including individual differences in neuroplasticity, adherence to the exercise program, and the severity of visual impairment. Thus, while we are optimistic about the potential impact of this combined intervention, the study will need to confirm these hypotheses through rigorous testing and analysis. Another limitation of this study is the lack of neuroimage description to verify the path of currents generated by stimuli and their effects on the central nervous system.

## Data Availability

The original contributions presented in the study are included in the article/Supplementary Material, further inquiries can be directed to the corresponding author.
